# Effects of auditing patient safety in hospital care: design of a mixed-method evaluation

**DOI:** 10.1186/1472-6963-13-226

**Published:** 2013-06-22

**Authors:** Mirelle Hanskamp-Sebregts, Marieke Zegers, Wilma Boeijen, Gert P Westert, Petra J van Gurp, Hub Wollersheim

**Affiliations:** 1 Institute of Quality Assurance and Patient Safety, Radboud University Nijmegen Medical Centre, Nijmegen, The Netherlands; 2 Scientific Institute for Quality of Healthcare (IQ healthcare), Radboud University Nijmegen Medical Centre, Nijmegen, The Netherlands

**Keywords:** Hospital, Patient safety, Safety management, Risk management, Complications, Management system audit, Clinical governance, Professional practice, Adverse events, Auditing

## Abstract

**Background:**

Auditing of patient safety aims at early detection of risks of adverse events and is intended to encourage the continuous improvement of patient safety. The auditing should be an independent, objective assurance and consulting system. Auditing helps an organisation accomplish its objectives by bringing a systematic, disciplined approach to evaluating and improving the effectiveness of risk management, control, and governance. Audits are broadly conducted in hospitals, but little is known about their effects on the behaviour of healthcare professionals and patient safety outcomes. This study was initiated to evaluate the effects of patient safety auditing in hospital care and to explore the processes and mechanisms underlying these effects.

**Methods and design:**

Our study aims to evaluate an audit system to monitor and improve patient safety in a hospital setting. We are using a mixed-method evaluation with a before-and-after study design in eight departments of one university hospital in the period October 2011–July 2014. We measure several outcomes 3 months before the audit and 15 months after the audit. The primary outcomes are adverse events and complications. The secondary outcomes are experiences of patients, the standardised mortality ratio, prolonged hospital stay, patient safety culture, and team climate. We use medical record reviews, questionnaires, hospital administrative data, and observations to assess the outcomes. A process evaluation will be used to find out which components of internal auditing determine the effects.

**Discussion:**

We report a study protocol of an effect and process evaluation to determine whether auditing improves patient safety in hospital care. Because auditing is a complex intervention targeted on several levels, we are using a combination of methods to collect qualitative and quantitative data about patient safety at the patient, professional, and department levels. This study is relevant for hospitals that want to early detect unsafe care and improve patient safety continuously.

**Trial registration:**

Netherlands Trial Register (NTR): NTR3343

## Background

Many patients face adverse events during their hospital stay. The occurrence of adverse events varies from 3% to 17% of all hospital admissions worldwide. A significant proportion of these adverse events result in death (5–21%), of which half could be prevented [1-7]. To obtain insight into safe hospital care, reliable data about the occurrence, causes, and preventability of adverse events have to be collected and made available. Commonly used methods for analyses of unsafe hospital care and improvement of patient safety include accreditation, external peer reviews, internal audits, patient safety systems, and performance indicators [8,9].

An internal audit should be an independent, objective assurance and consulting system for detecting patients’ risks of adverse events early, and it should encourage the continuous improvement of patient safety. An internal audit helps an organisation accomplish its objectives by providing a systematic, disciplined approach for evaluating and improving the effectiveness of risk management, control, and governance processes [10]. “Internal” means that trained employees of the hospital’s own organisation audit in one department, but work in another to guarantee some level of independent judgement. A major advantage of auditing is that, unlike registration of hospital data and mortality rates, it may also reveal the underlying causes of safety problems and could give clues to which improvements should be made to prevent adverse events. Auditing also ensures involvement of healthcare professionals in a peer–to-peer evaluation approach [11]. This bottom-up approach engages healthcare professionals at an early stage in the plan-do-check-act (PDCA) quality-improvement cycle.

Systematic literature reviews demonstrate that the effects of auditing and feedback on the behaviour of healthcare professionals and on patient outcomes range from none to substantial, with a maximum of a 70% increase in compliance with desired professional practice [12,13]. Audits in these reviews focused on improving professional practice and guideline adherence within the group of professionals responsible for patient care. Little is known about the effects of audits organised at the hospital level and directed at several levels of patient care, including policy, patient safety culture, guideline adherence of professionals, and outcomes at the patient level [12,13].

In order to be accredited, Dutch hospitals are required to have an internal audit system in place. All Dutch hospitals make efforts to control the quality and safety of care by means of some kind of auditing. These audits in the context of accreditation focus more on organisational preconditions and less on the behaviour of healthcare professionals and patient outcomes. However, whether these audits lead to early detection of risks of unsafe hospital care and, as a result, to safer healthcare, is unknown. Therefore, our study aims to evaluate the effects of auditing on patient safety outcomes and the performance of healthcare providers. Our main research questions are:

1. Does auditing improve patient safety outcomes and professional practice in hospitals?

2. What are the underlying processes and mechanisms of the effects of patient safety auditing?

The aim of this study protocol is to describe the study design. The effect of the audit system on various outcome measures and the process evaluation will be published in a separate manuscript.

## Methods/design

### Study design and setting

The study is a mixed-method evaluation study with a before-and-after study design.

The study is taking place in a 953-bed university hospital in the Netherlands. Eight hospital departments have been included; namely, general surgery, neurosurgery, obstetrics and gynaecology, orthopaedics, pulmonary medicine, general internal medicine, cardiology, and paediatrics. Audit procedures were or are planned for these departments from October 2011 until April 2013. The departments were selected because of the estimated high risks of preventable adverse events. The selected departments reasonably represent the medical practice in Dutch hospitals. Outpatient care and one-day hospital stays are excluded.

### Auditing of patient safety

We are evaluating the audit system of the Radboud University Nijmegen Medical Centre (RUNMC) because this hospital optimised their audit system (Table 1) after a disaster in 2006. In 2006 it became known that, in the previous 2 years, the RUNMC had a significantly higher mortality rate than the national average (6.7% versus 2.7%) for adult cardiothoracic surgery patients with cardiac failure [14]. The Dutch Health Care Inspectorate and the Dutch Safety Board reported that the factors of unsafe care consisted of the lack of the following: leadership, standards and protocols to deliver high-quality patient care, well-structured handovers, discussions about the mortality and complications rate, integrated care, and successful evaluation systems for patient safety [14,15].

**Table 1:** The development of an audit system

**Year**	**Steps for developing the audit system in the Radboud University Nijmegen Medical Centre**
2000	Introduction of an audit system for a test accreditation of hospital care from the Dutch Institute for Accreditation in Healthcare (NIAZ) → formal test of preconditions for good hospital care.
2002	The first accreditation from the NIAZ was achieved.
2006	The second accreditation from the NIAZ was achieved. However, despite the second accreditation, the Radboud case occurred. After the Radboud case, more focus on professional practice, leadership, team work, and patient safety outcomes were incorporated into the audit system. Valid and reliable instruments were selected to measure these aspects.
2009	An independent Institute for Quality Assurance and Patient Safety to monitor patient safety and quality of care was established.
	The audit process was professionalised:
	• The audit team must report to the Board of the Institute for Quality Assurance and Patient Safety instead of to the Board of Directors of the hospital.
	• The audit team was expanded with carefully selected physicians, nurses, and allied healthcare workers.
	• Extensive training for internal auditors to increasing the inter-rater agreement was set up.
	• The use of a reference framework made the audits more normative.
	• Follow-up: revisiting was implemented to examine the progress of patient safety.
2012	The audit system was optimised with:
	• Structural audit analyses.
	• Standard evaluation of experiences with auditing.

**Figure 1:**
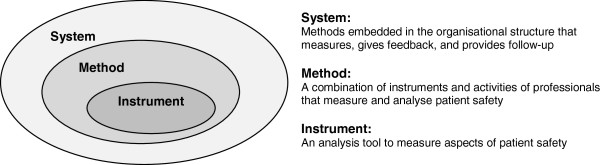
Audit components.

The RUNMC audit system is embedded in an organisational structure that measures, gives departmental feedback, and provides follow-up (Figure 1). This audit system combines professional activities and applied instruments to measure and analyse patient safety. The outcomes are compared to the legal, national, and professional standards of healthcare [16]. The methods used in the audit system are document studies, interviews, observations, surveys, medical record reviews, and appraisal and assessments (Table 2).

**Table 2:** Methods and instruments used within the audit system

**Audit system**	**Methods**	**Instruments**
Measurements	Studying policy and quality indicators	Online self-assessment tool based on legal, national, and professional practice standards [16]
	Semi-structured interviews of health care providers	Standardised interview forms [17]
	Systematic observations (e.g. physicians’ discussions of complications and patient handovers)	Standardised observation forms [17]
	Questionnaire about team functioning of healthcare providers	Team Climate Inventory [18]
Feedback of audit findings by presentation and report	Patient record review to measure adverse events	Standardised record review form based on a protocol originally developed by the Harvard Medical Practice Study [19]
	Assessment of the quality of medical and nursing patient records	Standardised assessment forms [19,20]
	Appraisal of document management (e.g. protocols and procedures) and guideline adherence	Standardised assessment forms partly based on the AGREE instrument [21]
Follow-up: revisiting 15 months after the audit to monitor improvements	Appraisal and assessment of quality of consultation and collaboration by main internal and external partners	Standardised appraisal and assessment questionnaire [22]

**Figure 2:**
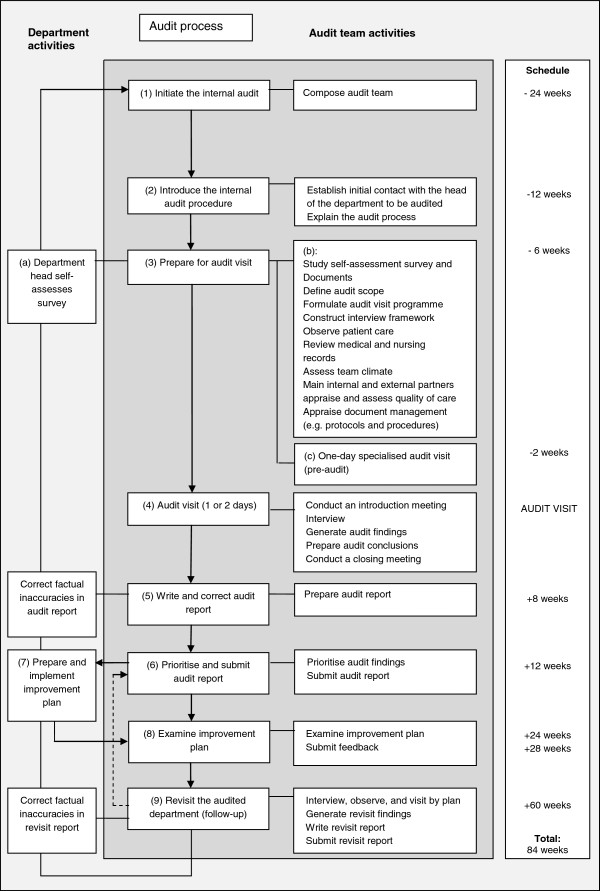
Audit steps and activities.

All of the 40 hospital departments that deliver or facilitate patient care are audited once every 4 years according to a fixed procedure and scheme. The auditing (Table 3) consists of nine steps (Figure 2). An independent Institute for Quality Assurance and Safety organises the audit procedures to ensure some kind of independence. To stress the independence of the institute: it is headed by a board of six representatives of physicians, nurses, allied healthcare workers, and healthcare researchers of the hospital. The audit team consists of five auditors, with at least one physician and one nurse. None have a direct relationship with the audited department. The chairman of the audit team is a medical department head of a contiguous specialism. Audit teams report audit findings to the board of the Institute for Quality Assurance and Safety. The board prioritises audit findings for the head of the audited department. The department head establishes and implements an improvement plan on the basis of the audit report and the prioritisation. The Board of Directors of the hospital controls the risk of unsafe care as indicated by means of the audit information and monitors the progress of improvement plans.

**Table 3:** Terminology

**Term**	**Definition**
Internal auditing	An independent, objective assurance and consulting activity designed to add value and improve an organisation's operations. It helps an organisation accomplish its objectives by bringing a systematic, disciplined approach to evaluating and improving the effectiveness of risk management, control, and governance [10]*.*
Audit process	A set of established methods for conducting the audit of a department. It describes the activities of auditors needed to achieve the audit objectives [23].
Audit team	A group of experienced, trained, and knowledgeable individuals selected to perform an internal audit. The audit team is responsible for auditing selected departments in its own hospital [23].
Auditee	The department or employee of the department being audited [23].

### Conceptual framework

**Figure 3:**
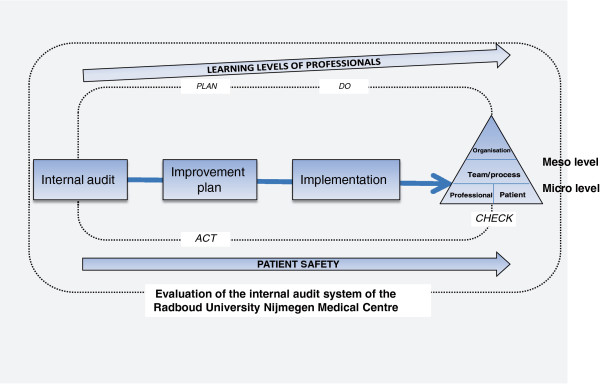
Conceptual framework.

For this evaluation study, we developed a conceptual framework (Figure 3) on the basis of theories from the field of quality improvement [24,25], implementation science [26], and Kirkpatrick’s learning model [27]. The conceptual model helps to explain the relationship between auditing and the possible effects on patient safety outcomes.

The PDCA cycle is a well-known model for continuous process improvement [28]. It can be operationalised as:

1. **Plan**: the department makes an improvement plan on the basis of the audit findings.

2. **Do**: the department implements the improvement plan.

3. **Check**: the audit team revisits to assess the progress of implementing improvement actions as ordered by the Board of Directors.

4. **Act**: the department takes action on the basis of the revisit findings. If the improvements cannot guarantee patient safety, the improvement plan should be adjusted.

In this study, we assume that internal audits affect Kirkpatrick’s four learning levels for healthcare providers and management [27]. The four levels of evaluation consist of:

• Level 1. **Reaction** – experiences of healthcare providers and management about auditing

• Level 2. **Learning** – the increase in knowledge and skills and the change in attitudes

• Level 3. **Behaviour** – change of behaviour as a result of learning

• Level 4. **Results** – the results in terms of a reduced level of adverse events after auditing.

### Outcomes

We measure several outcomes at the patient, professional, and department levels (Table 4). The primary outcomes are adverse events and complications. The secondary outcomes are patient experiences, the standardised mortality rate (SMR), prolonged hospital stay, team climate, and patient safety culture. These outcomes are assessed using medical record reviews, questionnaires, routine hospital administrative data, and observations. We collect data 3 months before the audit and will collect data again 15 months afterwards. We measure patient experiences also 9 months after auditing; the SMR and prolonged hospital stay are generated from the routine administrative hospital data monthly.

**Table 4:** Methods and instruments for measuring the effects of auditing

**Outcome variable**	**Data source**	**Frequency (type) of measurement and sample size per measurement**	**Moments of measurement**	**Unit of analysis**
**Primary outcome**				
Adverse events and complications	Retrospective patient record review based on a protocol originally developed by the Harvard Medical Practice Study [19]	2 (before–and-after measurement) *n* = 400	- 3 months; + 15 months	Patient
**Secondary outcomes**				
Patient experiences	Consumer quality-index questionnaire [29] based on the Consumer Assessment of Healthcare Providers and Systems [30]	3 (before-and-after measurement) *n* = 800	- 3 months; + 9 months and 15 months	Patient
Standardised mortality rate	Routine administrative data of the hospital	Continuously (time series) *n* = 233*	Monthly	Patient
Prolonged hospital stay	Routine hospital administration data	Continuously (time series) *n* = 3268**	Monthly	Patient
Team climate	Team Climate Inventory [18]	2 (before and after measurement) *n* = 132***	- 3 months; + 15 months	Professional or team
Patient safety culture	Hospital Survey on Patient Safety Culture [31,32]	2 (before-and-after measurement) *n* = 132***	- 3 months; + 15 months	Professional
	Safety walk arounds [33,34]	2 (before-and-after measurement) *n* = 8	- 3 months; + 15 months	Department

*Number of patients in the eight departments who died in 2012.

**Number of patients with prolonged stay in 2012 in the eight departments.

***Average number of clinical healthcare providers per department.

#### ***Patient level***

We define an adverse event as an unintended injury that results in temporary or permanent disability, death, or prolonged hospital stay, and it is caused by healthcare management rather than by the patient’s underlying disease [35,36]. A complication is an unintended and unwanted event or state during or following medical specialist treatment that has an unfavourable effect on the health of the patient to such an extent that adjustment of the medical treatment is necessary or to the extent that irreparable harm has occurred [37]. We use a structured method of patient record review, based on a protocol originally developed by the Harvard Medical Practice Study [19], to measure the incidence of adverse events and complications. Five trained physicians with a minimum of 10 years of clinical experience review retrospectively and independently the patient records of the sampled admissions that are positive for one of more of the screening criteria [6]. They assess the occurrence, cause preventability, and responsibility of the specialty for the adverse events. Complications are registered according to the classification of surgical complications [37]. To assess the inter-rater agreement between pairs of physicians, 10% of the patient records are independently reviewed a second time. In the case of disagreement about the presence or absence of complications and adverse events, both reviewers consider and discuss both reviews and reconsider their reviews to obtain consensus [38]. If they fail to reach agreement, the first review is leading.

We measure the experiences of patients with hospital care with the consumer quality (CQ) index [29]. The CQ index, based on the Consumer Assessment of Healthcare Providers and Systems, is a standardised and valid methodology for measuring, analysing, and reporting the patient customer experience in healthcare [30]. The survey comprises one specific dimension of patient safety and the other 13 dimensions of the survey are more or less related to patient safety. The survey is posted to a random sample of discharged patients. Non-respondents receive one reminder.

We will measure two patient safety indicators: SMR and prolonged hospital stay. The SMR is a method for comparing mortality ratios over time, or between subpopulations, taking into account the differences in population structure [39]. The ratio is of the observed to expected deaths, conventionally multiplied by 100. A prolonged hospital stay is the actual hospital stay of a clinically admitted patient that is more than 50% longer than expected [40]. A prolonged hospital stay takes into account the fact that patient stays tend to become prolonged after complications [41]. The SMR and hospital stay data are generated from the routine administrative data of the hospital.

#### ***Professional and team level***

We use the Team Climate Inventory (TCI) to measure the climate of the various teams of healthcare providers. Good team climate is an important characteristic of successful healthcare teams in hospitals. Working in teams is essential to provide proper and safe care. The TCI is a valid, reliable, and discriminating self-report measure of the climate of a hospital team [17]. The TCI is based on a four-factor theory of team climate for innovation, and it assesses the factors, vision, participative safety, task orientation, and support for innovation in 13 subscales.

To measure the patient safety culture at the department level, we use the Hospital Survey on Patient Safety Culture (HSOPS) [31,32]. The HSOPS is a valid and reliable survey for assessing the patient safety culture in hospitals. The HSOPS measures 12 dimensions of patient safety culture (Table 5) on the basis of ideas of unsafely designed care processes or systems that increase the likelihood of the occurrence of adverse events.

**Table 5:** Twelve dimensions of the Hospital Survey on Patient Safety Culture

**Dimensions of patient safety culture **[31]**,**[32]	
1.	Teamwork across hospital departments
2.	Teamwork within departments
3.	Hospital handovers and transitions
4.	Frequency of event reporting
5.	Non-punitive response to error
6.	Openness of communication
7.	Feedback and communication about error
8.	Organisational learning – continuous improvement
9.	Supervisor/manager expectations and actions promoting patient safety
10.	Hospital management support for patient safety
11.	Staffing
12.	Overall perceptions of safety

#### ***Department level***

One-hour safety walk arounds are used for observing patient safety culture [33,34]. The safety walk around consists of a person literally walking around on the ward, using a standardised observation list (Table 6), and paying specific attention to patient safety. During the walk around, the participants talk with employees about any risk situations in order to correctly interpret the items on the checklist as ‘safe’ or ‘unsafe’. The participants in the safety walk arounds are two healthcare providers of the ward who are responsible for the quality assurance tasks and one internal auditor.

**Table 6:** Items of patient safety culture checked during the safety walks

**Topic **[33]**,**[34]	**Some of the 65 items**
Medication safety	Double check before administration of the drug (the right drugs and right doses to the right patient at the right time)
	Keep medication inaccessible to unauthorized persons
Infection prevention	Wash hands before and after treatment of the patient
	Not wearing hand or wrist jewellery
Environment	Reduce risks of patients falling
	Make leaflets easily accessible to patients
	Test whether alarm systems work
Protocols and procedures of care	Ensure that only up-to-date instructions for the protocols and procedures of care are available
	Ensure that protocols and procedures are accessible
Information security	Keep medical and nursing patient record inaccessible to unauthorized persons
	Keep conversations between healthcare providers confidential
Sterile medical aids	Keep packaging of sterile materials closed
	Sterile materials for which the expiration date has passed must always be removed
Medical devices	Monitor maintenance periodically
	Provide training before use
Patient identification	Ensure that patients wear an identification bracelet
	Ensure that demonstrable checking takes place before blood products are given
Food safety	Check that the nutrition assistant ensures fluid and/or nutritional balance is complete
	Ascertain the temperature of the hot meal before serving
Reserved procedures	Determine that nurses have been trained and examined for risky medical procedures
Overall safety	The department must be clean and tidy
	Clean desk policy must be maintained in the reception room

### Sample size and precision calculation

With a sample of 50 medical records of patients from each of the eight hospital departments, we can estimate the difference in preventable adverse events and complications before and after auditing with a precision of 7%. The precision calculation of this study is based on Zegers et al. [42] and accounts for clustering in the before-and-after measurements. Since the patient records were drawn from the same departments, there will be some similarity in the before and after groups, i.e. a correlation between the before group and the after group.

### Statistical analyses

During the data collection, data are checked on a regular basis to identify out-of-range answers, inconsistent responses, and missing data. We will use SPSS version 20.0 for data analysis. Descriptive statistics will be used to describe baseline characteristics of patients, professionals, and departments.

#### ***Patient level***

A logistic regression analysis will analyse changes in the rates of patient admissions with adverse events and complications between before-and-after measurements, while correcting for clustering on the hospital department level. The intra-class correlations, the ratio of the between group, and the total variance will be calculated. Since the patient records will be different in the before–and-after measurements, the effect of timing (i.e. auditing) will be accounted for with a fixed effect for time (dummy variable for the second measurement). The incidence rates of adverse events and complications will be calculated with 95% confidence intervals (CIs). The inter-rater agreement within pairs of physicians will be expressed as a kappa (K) statistic with 95% CIs and as a percentage of records that agree about the presence or absence of complications and adverse events.

A linear mixed model will be used to analyse the before-and-after outcomes of the quality of hospital care according to the patients. To account for the possibility that changes over time will be influenced by changes in patient mix, terms will be added to the model for age, sex, education, and self-reported general and mental health status. Since the patients will be different in the before-and-after measurements, the effect of the timing of the auditing will be accounted for with a fixed effect for time (dummy variable for the second and third measurements).

We will use a linear mixed model with a Poisson distribution for the SMR and prolonged hospital stay to compare the rates from baseline to the end of the initial 18-month evaluation period (3 months before and 15 months after auditing). We will adjust this model for patient and departmental characteristics.

#### ***Professional and team level***

To account for the influence of department type on team climate and patient safety culture, we will analyse the before-and-after outcomes using a linear mixed model with the department type as a random effect. Since the professionals are almost the same in the before-and-after measurements, the difference scores in team climate and patient safety culture will be considered as dependent variables.

#### ***Departmental level***

The paired *t*-test (by department) will be used on the percentage of observed patient safety data collected during safety walk arounds to measure difference in patient safety on departments before and after auditing.

### Process evaluation

We will carry out the process evaluation to explain the effects. We will determine the components of internal auditing or factors other than auditing that could have influenced patient safety. We will document the different audit steps and the participation level of the healthcare providers (physicians, nurses, and allied healthcare workers) and department leaders. We will also measure the experience of these healthcare providers and department leaders with the auditing (level 1 of Kirkpatrick’s theory [27]) in an online survey, which has been tested and reviewed by experts for face validity. The survey will be e-mailed to those who participate in the audit within 2 weeks after the audit. Non-respondents will receive one reminder. According to the Kirkpatrick’s theory, the degree of exposure is equal to the extent that healthcare providers and leaders are familiar with auditing and have learned during auditing (level 2) and the way they have adapted their behaviour to improve patient safety (level 3). Semi-structured interviews will be used to measure the level of learning, behaviour change, and the related facilitators and barriers. Purposive sampling will be used to select a varied group of healthcare providers and department leaders [43]. We expect that 24 interviews will be enough to reach the point of theoretical saturation. If not, additional interviews will take place until no new information about the experiences with the audit system appears. An interview guide will be developed to facilitate these interviews. The interviews will be audio-taped and transcribed according to a standardised format. Atlas.ti.6 will be used for the qualitative analysis. We will study any changes in the safety policy and the number and type of implemented safety interventions after the audits by analysing audit reports, improvement plans, and revisit reports.

### Ethical approval and principles

The Ethical Committee of the Radboud University Nijmegen Medical Centre (RUNMC) approved the study on 11 July 2011. It conforms to Dutch law and privacy regulations and was judged not to involve human-subject research. The manuscript was registered on 12 March 2012 in the Dutch Trial Registry for clinical trials. The Dutch Trial Registration record number is NTR3343.

The participation of hospital departments in the study is voluntary. The researcher gave an oral presentation and written information to each department head about the consequences of participating in the study. Written consent has been obtained from all participating departments of the hospital. The data will be separated from the names of the participants (departments, healthcare providers, and patients) and published anonymously. Each participant will be identified in the database with a number and an identity code. These codes are available only to the researchers and the research assistant.

## Discussion

We evaluate the effects of patient safety auditing in hospital care. This study is relevant for hospitals that intend to use early detection of unsafe care and to improve patient safety continuously. Our study provides a better understanding of how auditing can help improve patient safety.

This study has several strengths and limitations. Most patient safety interventions, including auditing, are complex interventions because they often consist of multiple parts (multicomponent) and aim at several levels of the health pyramid (multilevel) and various actors (multitargeted) [44]. Therefore, we set up a mixed-method evaluation study in which we combine qualitative and quantitative measurement instruments to measure several outcomes at different levels: the patient, professional, and departmental levels. We use widely applied and thoroughly studied instruments to measure adverse events, patient experiences, team work, and patient safety culture among healthcare providers [5,18,32,45].

The causal relationship between internal auditing and change in adverse event rates is difficult to identify because of the presence of other hospital initiatives to improve patient safety [44,46]. Therefore, we will carry out a process evaluation to analyse the mechanism and processes responsible for the outcomes of the effect evaluation. We use questionnaires and interviews to study the factors that facilitate or hinder the effectiveness of auditing. This is also important for giving recommendations to other hospitals that want to implement some or all of our audit system.

We will analyse time series data of SMRs and prolonged hospital stay for better evidence of cause-and-effect relationships. Possible underlying secular trends will be detected, and perhaps they will help explain some of the effects [47].

To improve the internal validity by reducing bias, the following actions are taken. To reduce selection bias, patients were randomly selected from the hospital database. All the care personnel in the department who are working in clinical care are invited to fill in the TCI and HSOPS. To prevent participant recall bias, we survey patients 3 months after hospital admission at the latest. The experiences of healthcare personnel and department leaders with the audit system are measured within 2 weeks after the auditing (survey) and 1 month after the revisiting (interviews).

To decrease response bias, non-respondents receive a reminder. Missing values in the medical record review are checked. Inter-rater agreement of retrospective assessments is measured and given back to the physician reviewers to improve the reliability of the assessment of adverse events and complications.

Information bias is reduced by having a research assistant enter the research data. The data will be separated from the names of the participants (departments, healthcare providers, and patients). Each participant will be identified in the database with a number and an identity code. These codes will be available only to the research assistant and researchers. An independent researcher will check the data entries. An independent statistician will check the analyses.

To prevent confounding bias, we will study potentially confounding determinants of the relationship of auditing and patient safety by means of regression analysis and time series analyses.

To prevent publication bias, the study has been registered in the Dutch register for clinical trials. Additionally, we have installed an external Advisory Board with the special tasks of checking the integrity of the study group and giving methodological feedback.

The generalisability of the study will be low because it is carried out in a single hospital in a before-and-after design without concurrent controls. Ideally, the evaluation of the effects of auditing on hospital care should be studied in a large, multicentre trial with randomisation of hospitals with and without internal auditing [46,47]. Randomised controlled trials are the gold standard for evaluating healthcare interventions. A randomised, controlled, multicentre trial is not possible in this case. All Dutch hospitals have a legal obligation to systematically monitor, control, and improve the quality of care. Therefore, each one has some kind of auditing. However, for a hospital to temporarily refrain from a systematic analysis of quality assurance is unethical and illegal. Additionally, each hospital is unique in its management structure and the way internal auditing is performed. Therefore, a comparable control hospital with exactly the same management structure, organisation, and patient safety culture would be nigh impossible to find. We therefore decided to limit the evaluation of the effects of auditing on patient outcomes and professional practice to the one hospital that the other Dutch university hospitals regard as having the best practice for internal auditing [48]. Nevertheless, before-and-after designs are useful for studies that are part of local quality improvement projects such as audits, PDCA cycles, or action research [49]. In our circumstances, an evaluation of a local initiative in a before-and-after design is the only feasible option. This way of evaluating patient safety auditing with several measures and methods at different levels will provide the scientific proof we need for improving patient safety in hospitals.

## Abbreviations

Plan–Do–Check–Act (PCDA): is associated with the US statistician, educator, and consultant W. Edwards Deming, 1900–1993.

## Competing interests

All authors work in the RUNMC of which the audit system has been evaluated.

## Authors’ contribution

MZ, HW, WB, and PvG conceived the design of the study, drafted the initial research proposal and helped draft the final manuscript. MH acquired the data, wrote the first draft of the manuscript, and is responsible for the revisions. GW participated in the design and helped draft the manuscript. All authors read and approved the final manuscript.

## Pre-publication history

The pre-publication history for this paper can be accessed here:

http://www.biomedcentral.com/1472-6963/13/226/prepub
